# Prognostic Significance of miRNA Subtypes in Melanoma: A Survival Analysis and Correlation with Treatment Response Across Patient Stages

**DOI:** 10.3390/biomedicines12122809

**Published:** 2024-12-11

**Authors:** Mihaela Prodan, Alis Dema, Bianca Roxana Nataras, Edward Seclaman, Vlad Bloanca, Zorin Crainiceanu, Ilona Emoke Deak, Claudia Raluca Balasa Virzob, Ana-Olivia Toma, Roxana Manuela Fericean

**Affiliations:** 1 Doctoral School, “Victor Babes” University of Medicine and Pharmacy Timisoara, 300041 Timisoara, Romania; mihaela.prodan@umft.ro; 2 Department of Plastic Surgery, “Pius Brinzeu” Timis County Emergency Clinical Hospital, 300723 Timisoara, Romania; 3 ANAPATMOL Research Center, Department of Microscopic Morphology-Morphopathology, “Victor Babes” University of Medicine and Pharmacy Timisoara, 300041 Timisoara, Romania; dema.alis@umft.ro (A.D.);; 4 Department of Pathology, “Pius Brinzeu” County Clinical Emergency Hospital, 300723 Timisoara, Romania; 5 Department of Biochemistry and Pharmacology, “Victor Babes” University of Medicine and Pharmacy Timisoara, 300041 Timisoara, Romania; eseclaman@umft.ro; 6 Center for Complex Networks Science, “Victor Babes” University of Medicine and Pharmacy Timisoara, 300041 Timisoara, Romania; 7 Department of Plastic Surgery, “Victor Babes” University of Medicine and Pharmacy Timisoara, 300041 Timisoara, Romania; bloanca.vlad@umft.ro (V.B.); crainiceanu.zorin@umft.ro (Z.C.); sukosd.emoke@umft.ro (I.E.D.); virzob.claudia@umft.ro (C.R.B.V.); 8 Discipline of Dermatology, “Victor Babes” University of Medicine and Pharmacy Timisoara, 300041 Timisoara, Romania; manuela.fericean@umft.ro; 9 Department of Dermatology, Timisoara Municipal Emergency Hospital, 300254 Timisoara, Romania

**Keywords:** melanoma, microRNA, immunotherapy, biomarkers, oncology

## Abstract

Background and Objectives: Melanoma remains a leading cause of skin cancer mortality despite advancements in targeted therapies and immunotherapies. MicroRNAs (miRNAs) have emerged as potential biomarkers for cancer prognosis and treatment response. This study aims to analyze survival outcomes according to various miRNA subtypes, assess the association between specific miRNAs and treatment response, and include patient staging to evaluate their prognostic significance. Methods: A retrospective cohort study was conducted on 90 patients from the Pius Brinzeu County Emergency Clinical Hospital, Timisoara, between 2019 and 2022. The cohort included 45 patients with advanced-stage melanoma and 45 with benign nevi. miRNA expression levels were quantified using the miRNeasy Kit and the Human Cancer PathwayFinder miScript miRNA PCR Array. Survival analysis was performed using the Kaplan–Meier method, and Cox proportional hazards models were used to assess the impact of miRNA expression on survival. Logistic regression analyzed the association between miRNA markers and treatment response, adjusting for patient staging. Results: Elevated levels of hsa-miR-200a-3p and hsa-miR-335-5p were significantly associated with poorer overall survival (*p* < 0.01), particularly in stage III and IV patients. Conversely, higher expression of hsa-miR-451a correlated with improved survival rates (*p* = 0.02). Patients with increased hsa-miR-29b-3p expression showed a better response to immunotherapy (OR = 2.35, 95% CI: 1.15–4.79). Multivariate analysis confirmed that miRNA expression levels and patient staging were independent predictors of survival and treatment response. Conclusions: Specific miRNA subtypes are significant prognostic markers in melanoma, influencing survival outcomes and treatment responses across different patient stages. Incorporating miRNA profiling into clinical practice could enhance personalized treatment strategies and improve patient prognoses.

## 1. Introduction

Melanoma is one of the most aggressive forms of skin cancer, accounting for the majority of skin cancer-related deaths worldwide. Recent data indicate that while melanoma constitutes only about 1% of skin cancer cases, it is responsible for a disproportionate number of skin cancer fatalities [1,2,3]. Despite advances in early detection and the development of targeted therapies and immunotherapies, the prognosis for advanced-stage melanoma remains dire, with a five-year survival rate below 25% for metastatic cases [4,5,6]. The complex nature of melanoma, characterized by its ability to metastasize early and resist conventional therapies, underscores the urgent need for more effective diagnostic and therapeutic strategies.

The understanding of melanoma at the molecular level has significantly expanded in recent years, revealing a multitude of genetic and epigenetic alterations driving its development. Innovations such as the identification of BRAF mutations have led to targeted therapies that have improved outcomes for a subset of patients [7]. However, the heterogeneity of melanoma and its ability to develop resistance to treatments necessitate ongoing research into more reliable prognostic markers and personalized therapeutic approaches.

MicroRNAs (miRNAs) are small, non-coding RNA molecules that regulate gene expression at the post-transcriptional level and play pivotal roles in various cellular processes, including proliferation, apoptosis, and differentiation [8,9]. The dysregulation of miRNAs has been implicated in the initiation and progression of numerous cancers, including melanoma [10,11,12]. Specific miRNAs can function as oncogenes or tumor suppressors, influencing tumor behavior and patient outcomes.

Recent advancements have underscored the potential of miRNAs as biomarkers for cancer prognosis and predictors of treatment response [13,14,15]. In melanoma, certain miRNAs have been associated with tumor aggressiveness, metastatic potential, and resistance to therapies [16,17,18]. However, the prognostic significance of miRNA expression levels across different patient stages and their relationship with survival outcomes and treatment responses remains underexplored.

A new frontier in melanoma research involves the use of miRNAs in conjunction with other biomarkers and modern imaging techniques to refine the staging process and predict treatment efficacy. Understanding the interplay between miRNA expression, patient staging, and treatment efficacy could provide valuable insights into melanoma progression and aid in the development of personalized therapeutic strategies [19,20].

This study aims to perform a comprehensive survival analysis according to various miRNA subtypes, investigate the associations between specific miRNA markers and response to treatment, and include patient staging to evaluate their prognostic significance in melanoma. By integrating miRNA profiling with clinical data, we hope to uncover novel therapeutic targets and enhance the precision of treatment regimens, ultimately improving outcomes for melanoma patients.

## 2. Materials and Methods

### 2.1. Study Design

This retrospective cohort study was conducted at the Pius Brinzeu County Emergency Clinical Hospital, Timisoara, Romania, spanning from January 2019 to December 2022. We selected a population of patients diagnosed with melanoma, focusing on those who underwent surgical excision of the primary tumor as part of their initial treatment approach. Primary tumor lesions were excised in the Plastic Surgery Department of Pius Brinzeu County Emergency Clinical Hospital, Timisoara, and histopathologically examined in the hospital’s Department of Pathology. The study was conducted according to the guidelines of the Declaration of Helsinki and approved by the Ethics Committee of “Victor Babes” University of Medicine and Pharmacy, Timisoara, on 27 January 2022 (code 280). All participants provided informed consent, affirming their voluntary participation and understanding of the research purposes, procedures, and potential risks involved.

Based on our previous study results [21], we selected four specific miRNAs—hsa-miR-200a-3p, hsa-miR-335-5p, hsa-miR-451a, and hsa-miR-29b-3p—based on significant findings from prior research. These miRNAs were chosen due to their pronounced differential expression and association with critical clinical outcomes in melanoma. hsa-miR-200a-3p and hsa-miR-335-5p were significantly upregulated, suggesting a role in tumor progression, while hsa-miR-451a and hsa-miR-29b-3p were downregulated, indicating potential tumor-suppressive functions. Their distinct expression patterns and correlations with disease severity and prognosis make them particularly valuable for deeper investigation into their roles as biomarkers and their mechanistic implications in melanoma progression and treatment response.

### 2.2. Sample Collection

A total of 90 patients were included, comprising 45 patients with advanced-stage melanoma (stages III and IV) and 45 patients with benign pigmented nevi serving as controls. Inclusion criteria for melanoma patients were (1) histopathological confirmation of melanoma; (2) availability of formalin-fixed paraffin-embedded (FFPE) tissue samples; (3) complete clinical data, including staging and treatment records; (4) no prior chemotherapy or radiotherapy before sample collection. Patients were selected based on having received a uniform type of treatment, specifically immunotherapy with anti-PD1, as the most commonly used therapy in Romania at the time of the study, to ensure there were no differences in treatment types among the participants. Exclusion criteria included patients with other malignancies or autoimmune diseases, as well as cases of metastatic melanoma.

### 2.3. miRNA Purification and Analysis

miRNA was extracted from the FFPE tissue samples using the miRNeasy FFPE Kit from Qiagen, Maryland, USA, adhering strictly to the manufacturer’s protocols. The integrity and purity of the extracted miRNA were critically assessed using a NanoDrop spectrophotometer provided by Thermo Fisher Scientific, Waltham, MA, USA. For the purpose of profiling miRNA expression, we employed the Human Cancer PathwayFinder miScript miRNA PCR Array from Qiagen, which targets 84 miRNAs known to be associated with various cancers, including melanoma.

For the miRNA extraction process using the miRNeasy FFPE Kit from Qiagen, we began with deparaffinization of the FFPE sections through xylene treatment, followed by ethanol washes to effectively remove paraffin. Lysis was enhanced by prolonging incubation times beyond the standard protocol, followed by mechanical homogenization to ensure complete tissue digestion. During the RNA binding phase, we increased the ethanol volume to enhance miRNA adherence to the column and incorporated rigorous washing steps, adding an extra wash to remove potential inhibitors and contaminants.

For elution, the elution buffer was preheated to 50 °C to improve RNA recovery efficiency. Regarding controls, we implemented a multi-level control strategy: a synthetic miRNA was used as an external positive control to monitor extraction efficiency, no-template controls ensured there was no contamination across batches, and a synthetic miRNA spike-in control was added prior to extraction to assess process efficiency and compensate for any potential loss of RNA during the steps. These optimizations and controls were critical for ensuring the reliability of our miRNA quantification and were pivotal for our analysis of their roles in melanoma progression.

### 2.4. Data Analysis

Real-time PCR data were processed using the QIAGEN GeneGlobe Data Analysis Center online, with a threshold of two set for changes in miRNA expression in melanoma samples. This threshold was established to effectively discern significant shifts in miRNA levels related to melanoma, filtering out minimal background variations. By setting this criterion, only miRNAs demonstrating a minimum twofold increase or decrease in expression are considered significant. This approach aims to concentrate on alterations that hold potential biological and clinical relevance, thereby enhancing the reliability of the data by minimizing false positives that may arise from minor variations in miRNA expression, which are less likely to influence melanoma progression or detection.

In our real-time PCR assays for miRNA quantification, we employed an initial activation at 95 °C for 10 min, followed by 40 cycles of denaturation at 95 °C for 15 s and combined annealing/extension at 60 °C for 30 s. We used SYBR Green dye for detection, which binds to double-stranded DNA and emits fluorescence, allowing for sensitive quantification and specificity confirmation via melt curve analysis post-amplification. This setup ensured accurate and efficient amplification of miRNAs, which is critical for our analysis of their roles in melanoma progression.

Survival data, including dates of diagnosis, treatment, death, or last follow-up, were meticulously collected from hospital medical records. The overall survival time was defined from the point of melanoma diagnosis to the date of death or last available follow-up. Treatment responses were evaluated using the Response Evaluation Criteria in Solid Tumors (RECIST) guidelines, which allowed us to categorize patients as responders or non-responders based on observable changes in tumor size and progression post-treatment.

### 2.5. Statistical Analysis

Statistical analyses were conducted using SPSS software, version 27.0, from IBM Corp., located in Armonk, New York, USA. Continuous variables were summarized using means and standard deviations, whereas categorical variables were presented as frequencies and percentages, representing data distributions. Logistic regression models were utilized to explore the association between miRNA expression levels and treatment responses, adjusting for potential confounders like patient staging. Statistical significance was set at a two-tailed *p*-value of less than 0.05, ensuring that the findings were not due to chance.

For the statistical analysis of survival data, we utilized the Kaplan–Meier method to estimate survival functions, and differences in survival rates between groups were examined using the log-rank test. Additionally, Cox proportional hazards models were employed to perform multivariate analyses, considering variables such as miRNA expression levels, patient staging, age, and gender, providing a more nuanced understanding of factors influencing survival and treatment efficacy in melanoma patients.

## 3. Results

### 3.1. Demographics and Clinical Characteristics

In the demographic and clinical characteristics of the patients studied, no significant differences were observed in age or gender distribution between the melanoma group and the control group, with *p*-values of 0.218 and 0.304, respectively. Melanoma patients had an average age of 53.28 years and a standard deviation of 11.47 years, compared to 50.76 years and a standard deviation of 9.38 years in the control group. Gender distribution showed a slightly higher proportion of males in the melanoma group (28 males vs. 17 females) compared to the control group (23 males vs. 22 females). Breslow thickness, which measures the depth of melanoma invasion, averaged 2.85 mm with a standard deviation of 1.24 mm in melanoma patients. The presence of ulceration, an important prognostic factor in melanoma, was significantly higher in the melanoma group at 55.56% compared to 11.11% in the control group, yielding a statistically significant *p*-value of less than 0.001 (Table 1).

The study revealed significant differences in the expression levels of specific miRNAs between melanoma patients and controls. Specifically, hsa-miR-200a-3p and hsa-miR-335-5p showed significant upregulation in melanoma patients, with mean expression levels of 2.76 and 3.12, respectively, compared to 1.32 and 1.58 in controls, achieving fold changes of approximately 2.09 and 1.97 with a *p*-value of less than 0.001 for both. Conversely, hsa-miR-451a and hsa-miR-29b-3p were significantly downregulated in melanoma patients, with mean expression levels of 0.88 and 1.05, respectively, compared to 1.65 and 1.89 in the control group, resulting in fold changes of 0.53 and 0.56 and a *p*-value of less than 0.001 for each, as seen in Table 2.

### 3.2. miRNA Levels

**Figure 1:**
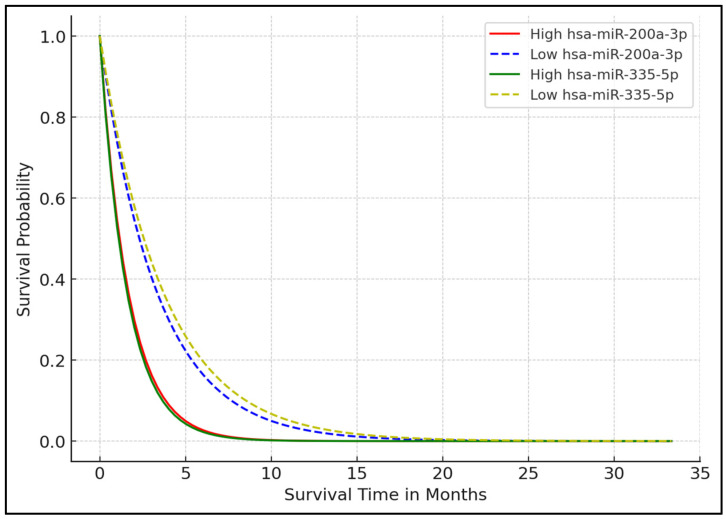
Kaplan–Meier survival analysis based on miRNA expression.

The Kaplan–Meier survival analysis presented in the study demonstrated significant variations in median survival times based on the expression levels of specific miRNAs in melanoma patients. Patients with high expression levels of hsa-miR-200a-3p and hsa-miR-335-5p exhibited considerably shorter median survival times of 18.75 and 17.62 months, respectively, compared to those with low expression, who showed median survival times of 26.48 and 27.30 months, respectively, with statistically significant *p*-values of 0.007 and 0.004. In contrast, high expression of hsa-miR-451a and hsa-miR-29b-3p was associated with longer median survival times of 28.56 and 27.89 months, respectively, versus 19.84 and 20.15 months for those with low expression, with *p*-values of 0.015 and 0.012, respectively. These findings indicate that high expression of hsa-miR-200a-3p and hsa-miR-335-5p may be adverse prognostic markers, while hsa-miR-451a and hsa-miR-29b-3p may serve as protective factors in melanoma prognosis, as seen in Table 3 and Figure 1.

### 3.3. Correlations Between miRNA Expression and Patient Outcomes

**Figure 2:**
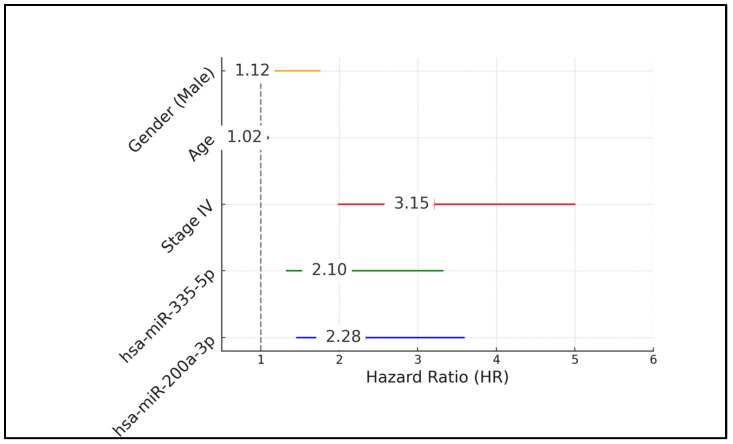
Forest plot analysis.

The multivariate Cox proportional hazards model analysis identified several independent predictors of poorer survival outcomes in melanoma patients. High expression levels of hsa-miR-200a-3p and hsa-miR-335-5p were significantly associated with increased risks of mortality, evidenced by hazard ratios (HRs) of 2.28 and 2.1, respectively, with corresponding *p*-values of 0.001 and 0.002. Notably, these miRNAs significantly contributed to the risk, suggesting their potential roles as biomarkers for aggressive melanoma behavior. Additionally, having stage IV melanoma was a strong independent predictor of poor prognosis, with a HR of 3.15 and a *p*-value of less than 0.001, underscoring the critical impact of advanced disease on survival. In contrast, age and gender (male) were not statistically significant predictors of survival, with HRs of 1.02 (*p* = 0.284) and 1.12 (*p* = 0.615), respectively, indicating that these factors did not influence survival outcomes as significantly as miRNA expression and disease stage in this cohort (Table 4, and Figure 2).

Higher expression levels of hsa-miR-29b-3p and hsa-miR-451a in responders, with means of 1.22 and 1.10, respectively, compared to non-responders who exhibited lower means of 0.88 and 0.76, were associated with better treatment responses, as evidenced by odds ratios (ORs) of 2.35 and 2.12, with *p*-values of 0.028 and 0.033, respectively. Conversely, elevated expression levels of hsa-miR-200a-3p and hsa-miR-335-5p were associated with poorer treatment outcomes. Responders had lower mean expressions of these miRNAs at 2.58 and 2.80, respectively, compared to non-responders, who showed higher means of 3.05 and 3.28. This association resulted in ORs of 0.65 and 0.68, with *p*-values of 0.041 and 0.047, respectively, indicating a significant link between higher expression of these miRNAs and reduced responsiveness to treatment (Table 5).

The analysis of miRNA expression levels across different stages of melanoma revealed significant differences, indicating a correlation with disease progression. For hsa-miR-200a-3p and hsa-miR-335-5p, expression levels were significantly higher in stage IV patients, with means of 3.12 and 3.42, respectively, compared to stage III patients who had lower means of 2.45 and 2.68, respectively. These differences were statistically significant, with *p*-values of less than 0.001. Conversely, hsa-miR-451a and hsa-miR-29b-3p showed lower expression levels in stage IV, with means of 0.72 and 0.88, compared to stage III, which had higher means of 1.02 and 1.18, with *p*-values of 0.002 and 0.001, respectively (Table 6).

The study assessed the correlation between miRNA expression and Breslow thickness, revealing significant relationships. hsa-miR-200a-3p and hsa-miR-335-5p demonstrated positive correlations with Breslow thickness, with correlation coefficients of 0.62 and 0.58, respectively, and *p*-values of less than 0.001 for both. This suggests that as the tumor depth increases, the levels of these miRNAs also rise, possibly indicating their roles in tumor progression or aggressiveness. In contrast, hsa-miR-451a and hsa-miR-29b-3p showed negative correlations with Breslow thickness, with correlation coefficients of −0.49 and −0.52, respectively, and *p*-values of 0.002 and 0.001. This indicates that higher tumor thicknesses are associated with lower levels of these miRNAs, suggesting their potential protective roles or differential regulation in more invasive stages of melanoma (Table 7).

The logistic regression analysis provided significant insights into the predictors of treatment response in melanoma patients. The coefficient for hsa-miR-29b-3p was positive (β = 0.85), indicating that higher expression levels of this miRNA significantly increase the likelihood of a positive treatment response, with an odds ratio (OR) of 2.35 and a confidence interval (CI) ranging from 1.15 to 4.79 (*p*-value = 0.028). This suggests that hsa-miR-29b-3p may play a beneficial role in the effectiveness of melanoma treatments.

Conversely, being at patient Stage IV was associated with a negative coefficient (β = −1.12), reflecting a decrease in the likelihood of a positive treatment response, with an OR of 0.33 and a CI from 0.17 to 0.64 (*p*-value = 0.001). This indicates a substantially lower probability of favorable response in advanced-stage melanoma. Similarly, higher expression of hsa-miR-200a-3p (β = −0.72) also correlates with a reduced probability of a positive response to treatment, with an OR of 0.49 and a CI from 0.25 to 0.95 (*p*-value = 0.041), as presented in Table 8.

## 4. Discussion

### 4.1. Literature Findings

This study demonstrates that specific miRNA subtypes have significant prognostic value in melanoma, affecting survival outcomes and treatment responses across different patient stages. The upregulation of hsa-miR-200a-3p and hsa-miR-335-5p in melanoma patients correlates with poorer survival and advanced disease stages, consistent with their potential roles as oncogenic miRNAs promoting tumor progression.

Conversely, the downregulation of hsa-miR-451a and hsa-miR-29b-3p is associated with improved survival and better treatment responses, suggesting their function as tumor suppressors. The negative correlation between these miRNAs and Breslow thickness further supports their protective role in melanoma. Also, the multivariate Cox regression analysis confirmed that miRNA expression levels, particularly hsa-miR-200a-3p and hsa-miR-335-5p, along with patient staging, are independent predictors of overall survival. This finding emphasizes the importance of incorporating miRNA profiling into prognostic assessments to enhance the accuracy of survival predictions and guide treatment planning [22,23,24,25].

The association between miRNA expression and treatment response highlights the potential of miRNAs as predictive biomarkers. Patients with higher levels of hsa-miR-29b-3p responded better to immunotherapy, suggesting that this miRNA could serve as a marker for selecting patients likely to benefit from specific treatments.

In the realm of microRNA (miRNA) research related to cutaneous malignant melanoma (CMM), two studies have provided significant insights. The study by Sand et al. [26] explored miRNA expression profiles in primary cutaneous malignant melanoma (PCMM), cutaneous malignant melanoma metastases (CMMM), and benign melanocytic nevi (BMN) through microarray analysis, identifying both previously known and novel miRNAs linked to CMM. This study validated 19 novel miRNA candidates, with several miRNAs such as hsa-miR-22, hsa-miR-130b, and hsa-miR-146b-5p showing upregulation, while others like hsa-miR-26a and hsa-miR-4291 were downregulated. These findings underscore the complexity and diversity of miRNA involvement in melanoma pathogenesis and progression. In a similar manner, the study by Polini et al. [27] focused on the tumor suppressor roles of hsa-miR-193a-3p and -5p in melanoma, revealing that their ectopic over-expression in melanoma cell lines substantially reduced cell viability and affected key signaling pathways involved in proliferation and apoptosis, such as Akt and Erk.

Similarly, in the study by De Tomi et al. [28], the therapeutic implications of Onconase (ONC), an amphibian secretory ribonuclease, were explored in the context of A375 melanoma cells. The research demonstrated that ONC treatment led to the upregulation of onco-suppressor microRNAs such as miR-20a-3p, miR-29a-3p, and miR-34a-5p, which corresponded to the downregulation of crucial onco-proteins involved in cell cycle regulation, survival pathways, and tumor metastatic potential, including cyclins D1 and A2, retinoblastoma protein, and cyclin-dependent-kinase-2. This regulation points to a potent anti-proliferative and anti-metastatic effect of ONC mediated through miRNA modulation. Similarly, Antonova et al. [29] identified significant miRNAs in stage IV melanoma patients and tested the anti-melanoma activity of various substances on a human melanoma cell line. Their results showed that microRNAs like hsa-miR-150-5p and hsa-miR-155-5p, which were upregulated in melanoma patients, could be effectively downregulated by treatment with humic substances, indicating a potential therapeutic role.

Moreover, the study by Sabato et al. [30] focused on developing a microRNA signature for melanoma detection via liquid biopsy, identifying a specific set of circulating microRNAs in plasma extracellular vesicles that could accurately distinguish melanoma patients from healthy controls. They achieved impressively high diagnostic accuracies with an area under the curve (AUC) of 1.00, 0.94, and 0.75 across three independent cohorts, also suggesting an immunosuppressive role for these microRNAs in melanoma. In a similar manner, the systematic review and meta-analysis by Wu et al. [31] consolidated data from multiple studies to evaluate the diagnostic value of circulating microRNAs for melanoma, demonstrating a robust overall diagnostic accuracy with a pooled sensitivity of 0.87, specificity of 0.81, and an AUC of 0.90.

In the study conducted by Nicholas Jones and Taichiro Nonaka [32], a systematic review and meta-analysis were performed to evaluate the diagnostic capabilities of circulating microRNAs (miRNAs) for melanoma. They analyzed nine studies involving 898 patients and found that circulating miRNAs had a high diagnostic accuracy with a sensitivity of 0.89 and a specificity of 0.85, accompanied by a diagnostic odds ratio of 45 and an area under the curve (AUC) of 0.93, highlighting their potential as reliable biomarkers for melanoma detection. In a similar manner, the research by Susan R Pfeffer and colleagues [33] focused on the detection of exosomal miRNAs in the plasma of melanoma patients, identifying specific miRNAs, such as miR-17 and miR-21, that were significantly upregulated in patients with metastatic melanoma compared to familial melanoma patients and unaffected controls. This study not only supported the use of miRNAs as biomarkers for melanoma but also suggested their potential role in differentiating between metastatic and familial forms of the disease, thereby offering insights into the underlying mechanisms of tumor progression and metastasis.

The clinical implications of our findings are profound, particularly in enhancing the precision of melanoma management. The identified miRNAs—hsa-miR-200a-3p, hsa-miR-335-5p, hsa-miR-451a, and hsa-miR-29b-3p—could serve as biomarkers for early detection and prognosis, offering a non-invasive method to assess tumor aggressiveness and potential treatment responses. For instance, elevated levels of hsa-miR-200a-3p and hsa-miR-335-5p might indicate a higher likelihood of poor prognosis and could guide oncologists in intensifying treatment strategies earlier in the disease course. Conversely, the presence of hsa-miR-451a and hsa-miR-29b-3p at higher levels could suggest a better prognosis and may support decisions to opt for less aggressive treatment protocols, reducing the burden of over-treatment. Thus, integrating these miRNA profiles into clinical practice could significantly refine personalized treatment plans, enhancing both efficacy and patient quality of life.

### 4.2. Study Limitations

The study’s retrospective design and relatively small sample size may limit the generalizability of the findings. Additionally, the use of FFPE samples might affect miRNA stability, although standardized protocols were employed to mitigate this issue. Future prospective studies with larger cohorts are warranted to validate these results. Moreover, future studies should evaluate exosomal miRNA levels, as these reflect the tumor microenvironment and can be detected non-invasively in blood, providing critical insights for understanding cancer progression and enhancing personalized treatment strategies. We also acknowledge the importance of capturing dynamic changes in miRNA expression over the course of treatment. For future studies, we recommend collecting serial blood samples or tumor biopsies from patients at baseline, during treatment, and post-treatment. This approach would enable monitoring changes in miRNA expression, potentially identifying specific miRNAs that could predict early treatment response or relapse. Based on our findings, we suggest incorporating single-cell RNA sequencing to analyze both miRNA and mRNA profiles within individual cells of melanoma tissue samples. This method would reveal how miRNA expression correlates with gene expression in different cell populations, enhancing our understanding of their impact on tumor biology and treatment response, particularly in distinguishing the roles of miRNAs in melanoma cells versus immune cells.

## 5. Conclusions

This study identified specific miRNA subtypes as significant prognostic markers in melanoma, influencing survival outcomes and treatment responses across different patient stages. The integration of miRNA profiling into clinical practice could enhance personalized treatment strategies, allowing for better patient stratification and improved prognoses. Further research is needed to validate these findings and explore the therapeutic potential of targeting these miRNAs.

## Figures and Tables

**Table 1:** Demographic and clinical characteristics of patients.

Variables	Melanoma Patients (n = 45)	Control Group (n = 45)	*p*-Value
Age (years)—mean ± SD	53.28 ± 11.47	50.76 ± 9.38	0.218
Gender (Male/Female)	28/17	23/22	0.304
Stage III/IV	26/19	N/A	N/A
Breslow Thickness (mm)	2.85 ± 1.24	N/A	N/A
Ulceration Present (%)	55.56%	11.11%	<0.001

SD—Standard Deviation.

**Table 2:** miRNA expression levels in melanoma patients vs. controls.

miRNA	Melanoma Mean ± SD	Control Mean ± SD	Fold Change	*p*-Value
hsa-miR-200a-3p	2.76 ± 0.35	1.32 ± 0.28	↑2.09	<0.001
hsa-miR-335-5p	3.12 ± 0.42	1.58 ± 0.31	↑1.97	<0.001
hsa-miR-451a	0.88 ± 0.22	1.65 ± 0.26	↓0.53	<0.001
hsa-miR-29b-3p	1.05 ± 0.25	1.89 ± 0.34	↓0.56	<0.001

SD—Standard Deviation; Interpretation: significant upregulation of hsa-miR-200a-3p and hsa-miR-335-5p, and downregulation of hsa-miR-451a and hsa-miR-29b-3p were observed in melanoma patients compared to controls. ↑ —Upregulation; ↓ —Downregulation.

**Table 3:** Kaplan–Meier survival analysis based on miRNA expression.

miRNA	High Expression Median Survival (Months)	Low Expression Median Survival (Months)	Log-Rank *p*-Value
hsa-miR-200a-3p	18.75 ± 2.14	26.48 ± 3.05	0.007
hsa-miR-335-5p	17.62 ± 2.08	27.30 ± 3.12	0.004
hsa-miR-451a	28.56 ± 3.22	19.84 ± 2.57	0.015
hsa-miR-29b-3p	27.89 ± 3.18	20.15 ± 2.60	0.012

Interpretation: patients with high expression of hsa-miR-200a-3p and hsa-miR-335-5p had significantly shorter median survival times; conversely, high expression of hsa-miR-451a and hsa-miR-29b-3p correlated with longer survival.

**Table 4:** Multivariate Cox proportional hazards model.

Variables	Hazard Ratio (HR)	95% Confidence Interval	*p*-Value
hsa-miR-200a-3p	2.28	1.45–3.60	0.001
hsa-miR-335-5p	2.1	1.32–3.33	0.002
Patient Stage (IV)	3.15	1.98–5.01	<0.001
Age	1.02	0.98–1.05	0.284
Gender (Male)	1.12	0.71–1.76	0.615

HR—Hazard Ratio; Interpretation: high expression of hsa-miR-200a-3p and hsa-miR-335-5p, along with stage IV disease, were independent predictors of poorer survival.

**Table 5:** Association between miRNA expression and treatment response.

miRNA	Responders Mean Expression ± SD	Non-Responders Mean Expression ± SD	Odds Ratio (OR)	*p*-Value
hsa-miR-29b-3p	1.22 ± 0.18	0.88 ± 0.21	2.35	0.028
hsa-miR-451a	1.10 ± 0.24	0.76 ± 0.19	2.12	0.033
hsa-miR-200a-3p	2.58 ± 0.31	3.05 ± 0.37	0.65	0.041
hsa-miR-335-5p	2.80 ± 0.36	3.28 ± 0.40	0.68	0.047

OR—Odds Ratio; Interpretation: Higher expression of hsa-miR-29b-3p and hsa-miR-451a was associated with better treatment response. Elevated hsa-miR-200a-3p and hsa-miR-335-5p levels were linked to poorer response.

**Table 6:** miRNA expression across patient stages.

miRNA	Stage III Mean ± SD	Stage IV Mean ± SD	*p*-Value
hsa-miR-200a-3p	2.45 ± 0.29	3.12 ± 0.38	<0.001
hsa-miR-335-5p	2.68 ± 0.33	3.42 ± 0.41	<0.001
hsa-miR-451a	1.02 ± 0.23	0.72 ± 0.19	0.002
hsa-miR-29b-3p	1.18 ± 0.20	0.88 ± 0.21	0.001

SD—Standard Deviation; Interpretation: miRNA expression levels varied significantly between stage III and stage IV patients, indicating a correlation between miRNA levels and disease progression.

**Table 7:** Correlation between miRNA expression and Breslow Thickness.

miRNA	Correlation Coefficient (r)	*p*-Value
hsa-miR-200a-3p	0.62	<0.001
hsa-miR-335-5p	0.58	<0.001
hsa-miR-451a	−0.49	0.002
hsa-miR-29b-3p	−0.52	0.001

Interpretation: Positive correlation between hsa-miR-200a-3p/hsa-miR-335-5p and Breslow thickness suggests higher miRNA levels, with increased tumor depth. Negative correlation for hsa-miR-451a/hsa-miR-29b-3p indicates lower miRNA levels with greater tumor thickness.

**Table 8:** Logistic regression analysis for predicting treatment response.

Variable	Coefficient (β)	Odds Ratio (OR)	95% Confidence Interval	*p*-Value
hsa-miR-29b-3p	0.85	2.35	1.15–4.79	0.028
Patient Stage (IV)	−1.12	0.33	0.17–0.64	0.001
hsa-miR-200a-3p	−0.72	0.49	0.25–0.95	0.041

Interpretation: higher hsa-miR-29b-3p expression increases the odds of a positive treatment response, while stage IV disease and elevated hsa-miR-200a-3p decrease the likelihood.

## Data Availability

Data are available upon request.
